# Supplementary material for: Do environmental, social, and governance scores improve green innovation? Empirical evidence from Chinese-listed companies
Source: PLoS One. 2023 May 25;18(5):e0279220. doi: 10.1371/journal.pone.0279220 (PMC10212161; doi:10.1371/journal.pone.0279220)
Supplement: Attachment — Submitted filename: PLOSE-Review.docx [file pone.0279220.s001.docx]

I thoroughly read the manuscript entitled "Do Corporate ESG Scores Improve Green Innovation? Empirical Evidence from Chinese Listed Companies" which I found interesting in term of title and novelty but before to publish the paper please incorporates my suggestion and comments.

1. Please do not use word abbreviations in the topic. Please write the full word "ESG".

2. The introduction is too weak in term of latest study. Please write more clearly about the mechanism that how ESG score improve green innovation.

3. Please include some latest literature

4. I am really astonished why the authors do not explain the base line method? please explain the details. For example, why we are using this methodology? advantages? comparing to another methodology.

5. The method which authors used in this paper is too much old but its ok to calculate the statics of the variables. Instead, authors can use CS-ARDL (which can calculate short and long run) Quantile and Quantile Approach, Quantile via movement.

6. Secondly author should use second generation panel unit root, otherwise the results will be called spurious regression. Most importantly authors should use cross-sectional dependence (CD) test (Most of economies and provinces are linked with trade relations, globalization, technology, knowledge transfer, and financial capital flows).

7. Please the discuss the results in more good way like why its negative, positive and significant? what is Economics behind is that?

8. Please check the English. Grammar and sentence.

Best of Luck
